# Magnetic resonance imaging thoracic organ-at-risk atlas for radiation oncology

**DOI:** 10.1016/j.phro.2026.100952

**Published:** 2026-03-21

**Authors:** Hannah Bainbridge, Michael J. Dubec, Andreas Wetscherek, Rob H.N. Tijssen, Jose Belderbos, Corine Van Es, David Cobben, Guido H.W. van Bogerijen, David Woolf, Marcel van Herk, Ferry Lalezari, Dow-Mu Koh, Fiona McDonald, Corrine Faivre-Finn

**Affiliations:** a Division of Imaging and Radiotherapy, The Institute of Cancer Research, London, UK; b The Royal Marsden NHS Foundation Trust, London, UK; c Department of Radiotherapy, Portsmouth University Hospital, Portsmouth, UK; d Division of Cancer Sciences, Faculty of Biology, Medicine and Health, The University of Manchester, UK; e Christie Medical Physics and Engineering, The Christie NHS Foundation Trust, Manchester, UK; f Joint Department of Physics, The Institute of Cancer Research and The Royal Marsden NHS Foundation Trust, London, UK; g Department of Radiotherapy, University Medical Center Utrecht, Utrecht, the Netherlands; h Department of Radiation Oncology, Catharina Hospital, Eindhoven, the Netherlands; i Department of Radiotherapy, The Netherlands Cancer Institute / Antoni van Leeuwenhoek, the Netherlands; j Department of Radiotherapy, Clatterbridge Cancer Centre NHS Foundation Trust, Liverpool, UK; k Department of Health Data Science, Institute of Population Health, University of Liverpool, Liverpool, UK; l Department of Clinical Oncology, The Christie NHS Foundation Trust and University of Manchester, UK; m Department of Radiology, The Netherlands Cancer Institute/Antoni van Leeuwenhoek, Amsterdam, the Netherlands

**Keywords:** Thoracic MRI, Atlas, Organs at risk, Lung cancer

## Abstract

**Background and purpose:**

The use of Magnetic Resonance imaging (MRI) for radiotherapy (RT) planning for locally advanced non-small cell lung cancer (LA NSCLC) could improve RT precision due to its superior soft tissue definition compared to computed tomography (CT). However, thoracic oncologists have limited experience of identifying thoracic structures on MRI. The aim of this study was to provide recommendations for MR sequences for thoracic organ at risk (OAR) contouring and present an atlas and descriptive instructions for delineation of thoracic OARs in the setting of MRI-guided radiation treatment planning and guidance.

**Materials and methods:**

MRI scans were acquired in nine patients with early-stage lung cancer on a diagnostic 1.5 Tesla system. MRI sequences included T_1_-weighted and T_2_-weighted imaging techniques, each optimised for visualisation of particular OARs. OAR delineations were carried out and reviewed by an international panel of thoracic oncologists and MR radiologists.

**Results:**

Thoracic MRI OAR contouring recommendations and atlas were developed by multi-institutional collaboration of six radiation oncologists and two MR radiologists. The atlas and contouring recommendations are described alongside high-resolution contoured MR images.

**Conclusions:**

This consensus MRI contouring atlas has a variety of potential applications, from integration of MRI within the standard CT-based workflow in order to improve the contouring accuracy of challenging structures such as the brachial plexus, to forming the foundation of an MRI-only workflow for use in MRI-guided treatment machines. This guideline should provide a useful reference for education and will facilitate uniformity in MRI-based contouring of OARs.

## Highlights

• An MRI thoracic OAR atlas and contouring recommendations are provided.
• MR sequences appropriate for OAR contouring are provided.
• Work aims to standardise OAR contouring for MR treatment planning and MR-guided workflows.

## Graphical abstract

**Figure:**
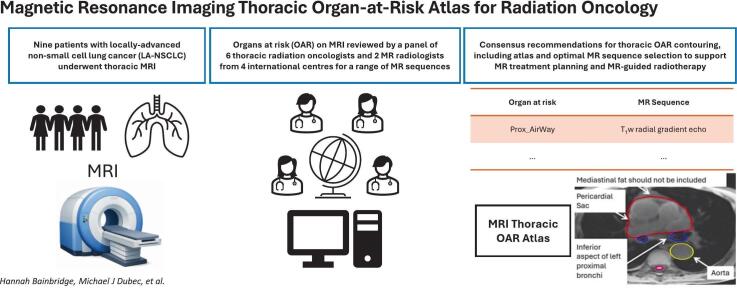

## 1 Introduction

Magnetic resonance imaging (MRI) has the potential to improve the radiotherapy treatment pathway for patients with thoracic malignancies [1]. One of the strategies to enhance radiotherapy treatment efficacy is personalisation and intensification: tailoring and increasing dose to each patient’s tumour whilst minimising dose to organs at risk (OARs). Accurate delineation of OARs is an essential component of this strategy. Discrepancies in OAR delineation can have significant dosimetric consequences [2], [3]. Furthermore, inaccuracies in OAR contouring can have clinical implications, particularly with treatment intensification [2], such as the increased risk of side effects including radiation-induced pneumonitis, cardiovascular events, and brachial plexus neuropathy [4].

With regards to the current computed tomography (CT)-based radiotherapy pathway, MRI may complement standard practice by providing improved soft tissue contrast, therefore enhancing the visualisation of OARs. CT-based contouring studies show significant inter- and intra-observer variations in OAR delineation [1], [3], [5], and for patients with thoracic malignancies, these differences are often most marked for the brachial plexus [6]. In addition, there are concerns about cardiac morbidity and mortality and an active area of research involves exploring radiation doses to cardiac sub-structures, which could be supported with the use of MRI. This is of particular concern for treatment intensification strategies [7].

With the availability of MRI-linear accelerator (MR-linac) technology [8] there is considerable interest in MRI-guided radiotherapy (MRgRT) workflows [9], which incorporate treatment planning, setup verification and motion monitoring, based on MRI information. Indeed MRgRT workflows are used in standard practice at many institutions to treat thoracic malignancies [1]. Such workflows, which include inter- and intra-fractional online adaption, may provide enhanced treatment personalisation through inter- and intra-fraction plan adaptation [1], [9].

The choice of MRI sequences for inclusion within RT workflows is important. Thoracic MR images are affected by the low proton density, and hence low signal intensity, in lung parenchyma and can also be affected by respiratory and cardiac motion related artefacts that must be minimised through motion management strategies [1]. The “on couch time” has to remain short for patient tolerability, large field sizes are required to cover whole thorax and geometrical accuracy has to be upheld [1].

In order to implement MRI into the RT planning stage, as well as within MRgRT workflows, physicians need to be able to identify and contour OARs and tumour on MRI [10]. However, unlike standard diagnostic ^18^F-FDG PET/CT and CT scans, MRI is not currently part of routine clinical workflow for patients with thoracic malignancies. Therefore, thoracic oncologists generally have limited experience in identifying structures on MRI. This has been used as an explanation to account for larger variability seen in MRI gross tumour volume (GTV) contouring in comparison to that seen with standard ^18^F-FDG PET/CT [11]. In CT-based practice, the use of a thoracic atlas for OARs has previously been shown to improve the consistency of contouring [3], and we anticipate that an MRI atlas will provide similar benefit.

The aims of this work were, (a) to provide guidance on MRI sequences appropriate for thoracic OAR contouring, and (b) to present a thoracic MRI atlas and practical descriptive guidance for delineation of OARs.

## 2 Methods

### 2.1 Image acquisition

Nine patients with early stage and locally advanced non-small cell lung cancer due to receive radical treatment with sequential or concurrent CRT or RT alone, were imaged, following written informed consent to local research ethics approved protocols (16/LO/0591, 17/NW/0334), at two UK institutions. MR imaging protocol and sequence development, which included prior sequence optimisation work [12], [13], was facilitated by the close collaboration between thoracic radiation oncologists, MR physicists and MR radiologists.

The dataset presented in this paper was acquired on a 1.5 Tesla (T) scanner (MAGNETOM Aera; Siemens Healthcare, Erlangen, Germany). All images were acquired without use of contrast, in the transverse plane as this is the traditional imaging plane used for thoracic CT delineation. Patients were positioned as for their standard of care CT planning scan, with arms up using a modified Extended Wing Board (Oncology Systems Limited) on a flat MRI tabletop. A coil bridge was placed anteriorly over the patient to suspend the anterior receiver coil to prevent deformation of the external patient contour [9]. A small flexible receiver coil was placed over a smaller curved bridge to improve signal over the anterior neck / upper chest. All images were acquired in free breathing.

MRI sequences included: (1) 3D T_1_-weighted (T_1_w) radial stack-of-stars fast gradient echo with fat suppression (T_1_w Radial GRE), (2) navigator-triggered T_2_-weighted (T_2_w) fast (turbo) spin echo (TSE) (T_2_w TSE non-fat-sat), and, one or both of, (3) T_2_w TSE with fat saturation (T_2_w TSE fat-sat) and/or (4) T_2_w TSE DIXON sequence, generating water-only and fat-only images. T_1_w Radial GRE data were used to generate 4D-MRI as described by Rank *et al* [14] using in-house developed software. MRI sequence parameters are provided in Supplementary Table S1.

4D-MR images were processed to generate mid-position (MidP) images via a deformable registration process as described by Wolthaus *et al* [15]. MidP CT, MidP T_1_w Radial GRE, and the T_2_w MR images were rigidly co-registered using a registration clipbox defined around the suggested OARs (Table 1). For T_1_w Radial GRE and T_2_w TSE (without fat saturation), the clipbox encompassed the whole thorax, whereas for T_2_w Dixon TSE, it covered the brachial plexus region. Registration accuracy was verified using anatomical landmarks, including the trachea, carina, aortic arch, and spinal canal, and the clipbox was adjusted as necessary to improve alignment for registration confidence.

**Table 1:** Thoracic organs at risk (OARs) with summary of contouring recommendations and optimal MR sequences for delineation. Comprehensive guidelines are provided in Section 3.

**Thoracic OAR**	**TMG-263 nomenclature**	**Contour recommendation summary**	**MRI sequence**
Trachea	Trachea	The trachea is contoured as a single structure extending from 2 cm above the carina to the inferior border of the cricoid cartilage. The distal 2 cm of the trachea is included within the proximal bronchial tree (Fig. 1). Tracheal cartilage is contoured anterior-laterally, with the posterior muscular wall included (Fig. 1, Fig. 2).	T_1_w radial stack-of-stars spoiled gradient echo (Radial GRE)
Proximal Airways	Prox_AirWay	Includes the distal 2 cm of trachea, carina, right main bronchus, upper, middle and lower lobe bronchi and bronchus intermedius, and left main bronchus, upper, lingual and lower lobe bronchi. Contouring ends at the bifurcation of the second-order bronchi (Fig. 1, Fig. 2).	T_1_w Radial GRE
Spinal Cord	SpinalCord	The spinal cord is contoured, rather than the bony extent of the spinal canal, from the inferior border of the cricoid cartilage. Contouring continues on every slice to the inferior aspect of the second lumbar vertebra. If a dorsal root ganglion is seen emerging from the cord, it is not included. The spinal cord appears hyperintense on T_1_-weighted radial GRE images and is distinguishable from the hypointense cerebrospinal fluid.	T_1_w Radial GRE
Pericardial Sac	Pericardium	The heart and pericardial sac are contoured as a single structure. The superior aspect of the heart (or base) is defined as the superior aspect of pulmonary artery, as seen on a coronal reconstruction, and extends inferiorly to the apex of the heart. Both pulmonary arteries are fully contoured above the main bronchus, while the pulmonary veins are excluded. When the inferior vena cava becomes distinctly separated from the heart, it is not included in the pericardial sac contour.	T_1_w Radial GRE
Great Vessels	GreatVes	The great vessel contours (aorta and vena cava, excluding the pulmonary artery or vein) include the vascular wall and muscular layers out to the fatty adventitia on all slices.	T_1_w Radial GRE
Oesophagus	Esophagus	The oesophagus is contoured from the inferior border of the cricoid cartilage to the gastro-oesophageal junction. The contour includes the mucosa, submucosa, and all muscular layers. The fatty adventitia cannot be distinguished from mediastinal fat and appears hyperintense on the T_2_-weighted images. The muscular layers demonstrate intermediate T_2_-weighted signal intensity and form the outer contour boundary of the structure.	T_2_w TSE without fat saturation
Brachial Plexus	BrachialPlexs	The contour includes the C7, C8, and T1 nerve roots and the brachial plexus caudal to these, including the trunks, divisions, and cords. After exiting the neural foramina, the C8 and T1 roots converge to form the inferior trunk, while C7 continues to form the middle trunk. The trunks lie posterior and superior to the subclavian artery. Progressing laterally, beyond the outer border of the first rib, the divisions then become cords, the principal cords from C7, and C8/T1 being the posterior and medial cord respectively (Fig. 5). Contour delineation is performed primarily on T_2_-weighted Dixon water images, with fat-only images used for confirmation. Myelinated nerve fibres appear hyperintense on water-only images, while bony structures appear hypointense due to fatty marrow. Where the nerves become indistinguishable from the subclavian artery, an envelope contour is applied, bounded medially by lung, laterally by the second rib, and anteriorly by the posterior aspect of the subclavian vein (Fig. 5).	T_2_w Dixon TSE

### 2.2 MRI OAR atlas generation

The OAR atlas was devised based on previous CT-based guidelines [16], [17], with adaptations to account for the improved soft tissue contrast on MRI. Thoracic OARs included the Trachea, Proximal Bronchi, Spinal Cord, Pericardiac Sac, Great Vessels, Oesophagus, and Brachial Plexus (list also provided in Table 1, along with TMG-263 naming convention).

Pre-treatment images from nine patients were reviewed by an international panel comprising six thoracic radiation oncologists and two MR radiologists, who met in person to identify the most appropriate MR sequences for thoracic OAR visualisation. Each expert independently contoured the OARs using ‘Big Brother’ software [18], after which all contours were jointly reviewed.

Following this workshop, pre-treatment imaging datasets from two of the nine patients were selected for contouring to generate the final atlas. These two pre-treatment patient datasets, with early-stage lung cancer, were chosen for the atlas as their small disease volume did not interfere with delineation of individual thoracic OARs. Contouring for the final atlas was carried out using Raystation (Raysearch Laboratories). To facilitate delineation, all images were individually windowed to optimise visibility of the OAR of interest, which was necessary due to potential differences in gain settings across MR acquisitions.

For the final atlas, initial contours were generated by a thoracic radiation oncologist and an MR radiologist from one institution followed by subsequent review and editing by a second thoracic radiation oncologist and second MR radiologist, from two separate international institutions. The final step was a review and approval of the contours and associated annotations by 4 radiation oncologists from 4 international institutions.

Data including anonymised images and radiotherapy structure sets can be obtained from the corresponding author upon reasonable request.

## 3 Results

Following the sequence review, contouring, and final review session, the findings and recommendations for OAR contouring are presented below, based on the sequences identified as optimal for each individual OAR.

Table 1 summarises the MR sequences selected for delineating each thoracic OAR. For example, the T_1_w Radial GRE sequence was chosen as it provided visualisation of OARs including trachea and proximal bronchi, and due to its radial stack-of-stars readout that can be acquired in free breathing, providing an approximate average respiratory position, with minimal motion artefacts and the ability for 4D image reconstruction [19]. It further provides an image contrast similar to that of CT. T_2_w TSE sequences were chosen for delineation of the oesophagus [20] and were acquired using a navigator trigger, and hence presented as end-exhale images [21]. The T_2_w Dixon-TSE water only and fat only images were chosen for delineation of the brachial plexus as they provide good contrast between nerve fibres and surrounding tissues [22], [23].

### 3.1 Oars on T_1_w radial GRE

#### 3.1.1 Trachea

**Fig. 1:**
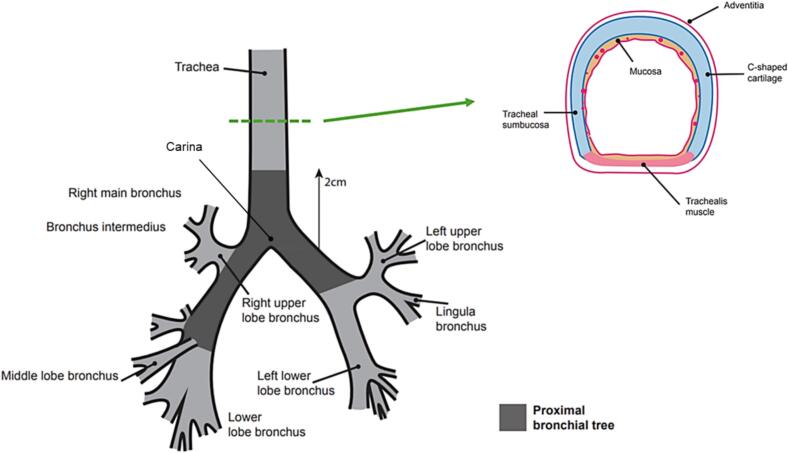
Illustration of the proximal bronchial tree anatomy with the insert highlighting the layers of the tracheal wall.

**Fig. 2:**
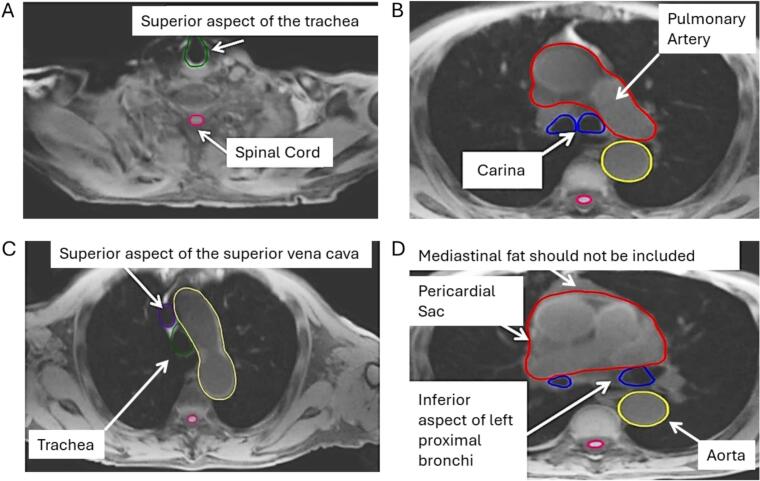
Example transverse T_1_w Radial GRE images annotated and contoured; with landmarks including: (A) superior aspect of trachea, (B) carina, (C) superior aspect of superior vena cava, and (D) inferior aspect of left proximal bronchi. The full atlas, accompanied with unannotated images, is provided in Supplementary Fig. S1. Pink contour: Spinal cord; Blue contour: Proximal bronchi; Green contour: Trachea; Red contour: Pericardial sac; Yellow contour: Aorta; Purple contour: Superior vena cava. (For interpretation of the references to colour in this figure legend, the reader is referred to the web version of this article.)

The trachea was contoured as one structure from 2 cm above the carina to the inferior border of the cricoid cartilage. The distal 2 cm of trachea was included in the proximal bronchial tree structure (Fig. 1). The cartilage of the trachea was included anterior-laterally and the muscular wall posteriorly (Fig. 1, Fig. 2).

#### 3.1.2 Proximal bronchi

The following structures were included: distal 2 cm of trachea, carina, right main bronchus, upper, middle and lower lobe bronchi and bronchus intermedius, and left main bronchus, upper, lingual and lower lobe bronchi. Contours ended immediately at the bifurcation of second order bronchi (Fig. 1, Fig. 2). A coronal view aided identification of the carina.

#### 3.1.3 Spinal cord

The spinal cord is clearly visible on T_1_w Radial GRE images and was contoured instead of the bony extent of the spinal canal, typically delineated in CT-based guidelines (Fig. 2) [16]. The spinal cord is hyperintense (bright) on T_1_w Radial GRE images and can be differentiated from cerebrospinal fluid which is hypointense (dark) [24]. The spinal cord was contoured starting at the inferior border of the cricoid cartilage. Contours were continued on every slice caudally until the inferior aspect of the second lumbar vertebra. If a dorsal root ganglion was seen emerging from the cord this was not included.

#### 3.1.4 Pericardial sac

The heart was contoured along with the pericardial sac as one structure. On T_1_w Radial GRE images the pericardium appears hyperintense (bright) (Fig. 2). The superior aspect of the heart (or base) was defined as the superior aspect of pulmonary artery, as seen on a coronal reconstruction, and extends inferiorly to the apex of the heart. Both pulmonary arteries are fully contoured above the main bronchus and the pulmonary veins are excluded**.** When the inferior vena cava (IVC) became distinctly separated from the heart it was not included in the pericardial sac.

#### 3.1.5 Great vessels

The great vessels (aorta and vena cava, but not the pulmonary artery or vein) were contoured on T_1_w Radial GRE images to include the vascular wall and muscular layers out to the fatty adventitia on all MR slices.

### 3.2 Oars on T_2_w TSE

#### 3.2.1 Oesophagus

**Fig. 3:**
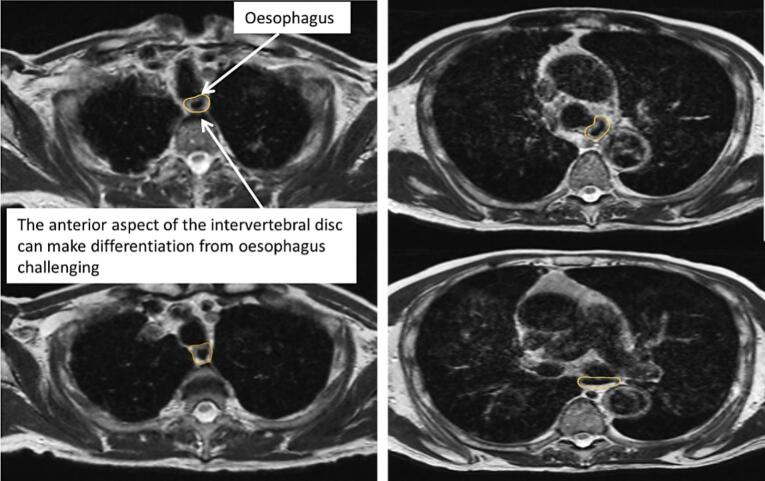
Example transverse T_2_w TSE (turbo spin echo) images highlighting oesophagus, from clavicular heads (top-left) to pulmonary artery bifurcation (bottom-right). Orange contour: Oesophagus. Additional images, accompanied with unannotated images, are provided in Supplementary Fig. S2. (For interpretation of the references to colour in this figure legend, the reader is referred to the web version of this article.)

The oesophagus was contoured from the inferior border of the cricoid cartilage to the gastro-oesophageal junction on the T_2_w TSE images (Fig. 3). The contours included the mucosa, sub-mucosa and all muscular layers [16]. The fatty adventitia cannot be distinguished from the mediastinal fat and appears hyperintense (bright) on T_2_w images. The muscular layers return intermediate T_2_w signal intensity (grey) and form the outer contour boundary of the structure.

### 3.3 Oars on T_2_w TSE dixon

#### 3.3.1 Brachial plexus

**Fig. 4:**
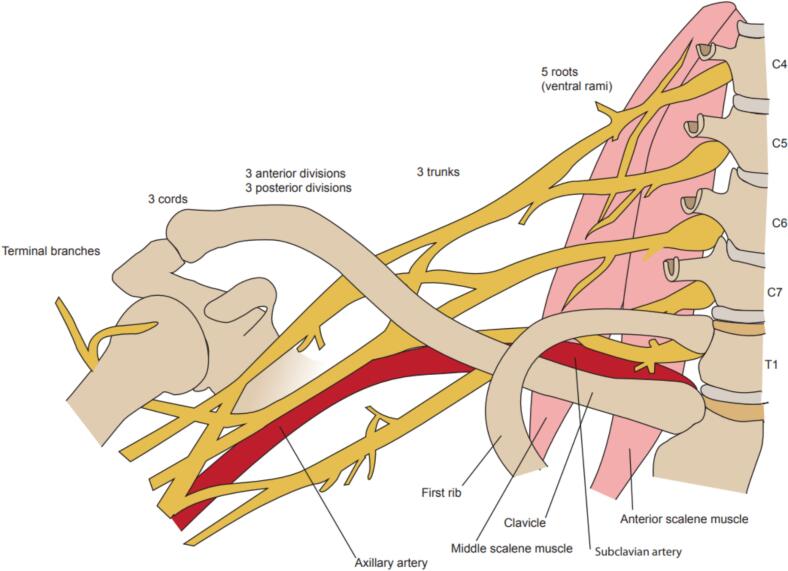
Illustration of brachial plexus anatomy indicating the position of the subclavian artery relative to the brachial plexus, and the anatomical differentiation of the brachial plexus from roots to trunks, divisions, cords and terminal branches. Additional illustration provided in Supplementary Fig. S3.

**Fig. 5:**
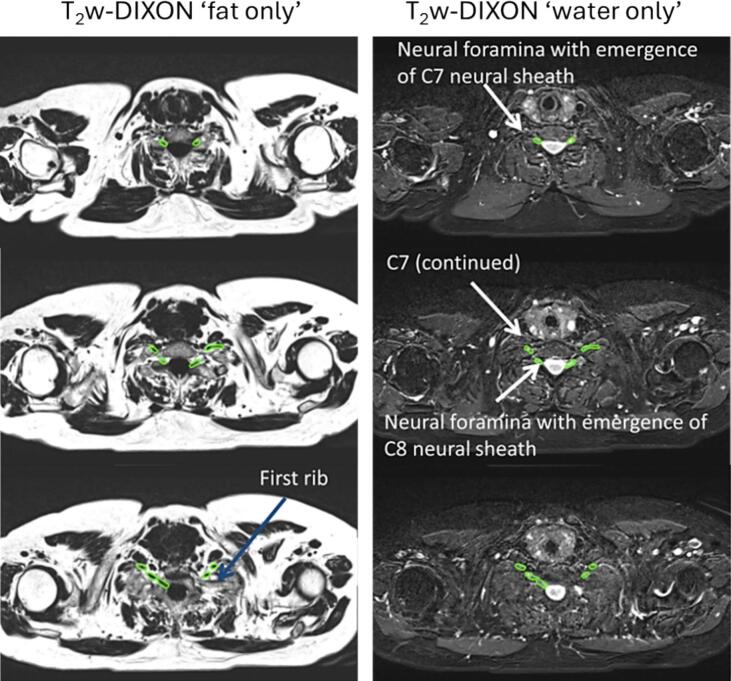
Transverse T_2_w Dixon TSE (T_2_-weighted Dixon turbo-spin echo) images of brachial plexus; (left column) T_2_w Dixon TSE (fat only) and (right column) T_2_w Dixon TSE (water only) for contouring the brachial plexus nerves. Green contour: Brachial plexus envelope. Additional images accompanied with unannotated images are provided in Supplementary Fig. S4. (For interpretation of the references to colour in this figure legend, the reader is referred to the web version of this article.)

For thoracic radiotherapy only the lower part of the brachial plexus is clinically relevant and thus was contoured in this atlas (Fig. 4, Fig. 5). Contouring was performed on the T_2_w Dixon TSE water images and included the C7, C8 and T1 nerve roots and brachial plexus structures caudal to this which included the trunks, divisions and cords.

After exiting the vertebral foramina, the C8 and T1 nerve roots converge to form the inferior trunk. C7 continues in a more direct trajectory to become the middle trunk, and on T_2_w Dixon TSE water images it is often possible to track the principal element of the C7 nerve from exiting the foramen to the superior posterior aspect of the subclavian artery which assists with orientation (Fig. 5). The trunks lie posterior and superior to the subclavian artery (Fig. 5) [25], [26].

Progressing laterally, beyond the outer border of the first rib, the divisions then become cords, the principal cords from C7, and C8/T1 being the posterior and medial cord respectively (Fig. 5). However, there are six additional branches from the posterior and medial cords [25], [26], [27]. The cords and branches are anatomically distributed around the subclavian artery, and are unlikely to all be visible on the T_2_w TSE Dixon water images, in which case a contour enveloping the subclavian artery is included, as is subsequently described.

T_2_w TSE Dixon water (i.e. ‘water-only contrast’) images were primarily used for delineation, with the ‘fat-only’ images providing additional information. Myelinated nerve fibres appear bright on water-only images and dark on fat-only images. Bony vertebrae and ribs, because they contain fatty marrow, appear hypointense (dark) on MRI.

The following steps were followed:

1. The neural foramina at the C6-C7 level were identified to locate the origin of the C7 nerve root. The neural sheath was contoured as it emerged from the neural foramina and extended infero-laterally towards the subclavian artery.
2. The C8 and T1 nerves were contoured until they reached the posterior aspect of the subclavian artery. As illustrated in Fig. 5, the C8 and T1 nerves converge as they progress from roots to trunks. In order to contour the C8 and T1 roots, the neural foramina above (C8 root) and below (T1 root) T1 were identified. The first rib was used to locate the T1 vertebra, and the sagittal reconstructed T_1_w Radial GRE images were used to help with orientation and vertebral position. Before using the first rib to aid orientation, it should be confirmed that the patient does not have a cervical rib.
3. At the level of the subclavian artery (when the nerve may become indistinguishable from the artery), the contour was extended to form an envelope. This envelope structure included the subclavian artery and was defined by the lung medially, the edge of the second rib laterally, and the posterior aspect of the subclavian vein anteriorly.

## 4 Discussion

Current thoracic OAR delineation atlases are CT-based, and have limitations, particularly in relation to the brachial plexus [6]. The implementation of MRI within treatment planning and MRgRT workflows, with incorporation of an MRI-based atlas for thoracic OAR delineation and education stand to improve inter-observer contouring reproducibility with the aim of ultimately reducing normal tissue toxicities. The atlas developed in this work provides guidance on thoracic OAR contouring on MRI which can be integrated into clinical trials evaluating MR-guided radiotherapy. Also important, are recommendations for contouring of GTV in lung cancer using MRI, which we have addressed separately [28].

The novel inclusion of MRI into an MRI-guided workflow represents a step-forward in radiation therapy. However, the application of this technology necessitates further learning and training for radiation oncologists who are accustomed to contouring on CT. Whilst the T_1_w Radial GRE (e.g. Siemens STARVIBE used here) provides similar image contrast to CT, the T_2_w TSE and T_2_w TSE-Dixon sequences demonstrate mediastinal structures and nerve sheaths in a complementary manner to CT. These sequences represent standard MR techniques that are broadly applicable across different systems, including varying field strengths and MR-Linac platforms; however, further optimization may be necessary to achieve optimal performance on these systems. These contouring recommendations provide a practical way of both acquiring and using information from MRI to contour thoracic OARs. For example, in relation to the brachial plexus, which is delineated with large inter-observer variability [3], [5], [6], despite a number of CT guidelines for delineation [16], [17], the advantage of soft tissue contrast on MRI allows a more precise definition for contouring. It is important to note that delineating on MRI could influence the OAR constraints compared to CT-based OAR delineation and will be an area of future research.

A limitation of this study is that contoured and annotated images are from only two patients and therefore does not account for individual anatomical variations, however patients with small volume disease were chosen to limit the effect of disease on a proposed standardised atlas. Another limitation is that the different sequences were acquired with different motion management strategies, limiting the ability to fuse the images with the standard 4D planning CT. The balance between image sequence quality and optimal motion management acquisition remains a challenge. Development of robust image deformation strategies going forwards may help overcome this. Advances in MR imaging have introduced techniques such as 3D stack-of-stars sequences, including balanced steady-state free precession (bSSFP)-type contrast, which have also been implemented on MR-Linac systems and that can be acquired during free-breathing [9]. While such sequences were not evaluated in the present study, they warrant future investigation, including potential confounding effects such as susceptibility artefacts, for their utility in OAR and lesion contouring. Nonetheless, the recommendations presented here remain applicable to these techniques, owing to the similarities in image contrast with the sequences assessed in this work.

In addition to its potential for use in standard CT-based planning to enhance personalisation of treatment through improved OAR delineation, the proposed atlas will also support MRI-guided radiotherapy workflows. Daily adaptive re-planning is one of the many potential benefits of an MRI-only workflow [1]. This pathway relies not only on the generation of daily MR images, or even synthetic CT [29], but also on appropriate contouring of tumour and OAR. Online manual contouring of tumour and OAR prior to every fraction of treatment is not a feasible workflow, but instead auto-segmentation or semi-auto-segmentation is likely to be used [30]. A number of techniques for auto-segmentation have been proposed, but the most robust of these uses a pre-existing library of contoured images to automatically generate contours on a new set of images [30], [31]. The ability to perform auto-segmentation using this approach is only as good as the quality of contours which have been included in the image contour library. This emphasises the importance of an MR atlas to standardise the approach to contouring and thus enable the building of a digital library of quality contoured images for training auto-segmentation software algorithms.

This atlas, developed through international collaboration of radiation oncologists and radiologists currently working in the field of thoracic MRI, has a diversity of uses. MRI can be integrated into a standard CT-based workflow to improve the contouring accuracy of challenging OARs such as the brachial plexus. In addition, MRI forms an integral part of an MRI-guided radiotherapy workflow and data generated as a result of using this delineation guideline can form the foundation of data which will be used to train auto-segmentation software.

## CRediT authorship contribution statement

**Hannah Bainbridge:** Writing – review & editing, Writing – original draft, Visualization, Resources, Project administration, Methodology, Investigation, Formal analysis, Data curation, Conceptualization. **Michael J. Dubec:** Writing – review & editing, Writing – original draft, Visualization, Software, Resources, Project administration, Methodology, Investigation, Formal analysis, Data curation, Conceptualization. **Andreas Wetscherek:** Writing – review & editing, Writing – original draft, Visualization, Software, Resources, Methodology, Investigation, Data curation, Conceptualization. **Rob H.N. Tijssen:** Writing – review & editing, Writing – original draft, Visualization, Software, Resources, Methodology, Investigation, Data curation, Conceptualization. **Jose Belderbos:** Writing – review & editing, Writing – original draft, Methodology, Investigation, Data curation, Conceptualization. **Corine Van Es:** Writing – review & editing, Writing – original draft, Methodology, Investigation, Data curation, Conceptualization. **David Cobben:** Writing – review & editing, Writing – original draft, Methodology, Investigation, Data curation, Conceptualization. **Guido H.W. van Bogerijen:** Writing – review & editing, Investigation. **David Woolf:** Writing – review & editing, Investigation. **Marcel van Herk:** Writing – review & editing, Writing – original draft, Visualization, Supervision, Software, Resources, Project administration, Methodology, Investigation, Formal analysis, Data curation, Conceptualization. **Ferry Lalezari:** Writing – review & editing, Writing – original draft, Visualization, Software, Resources, Project administration, Methodology, Investigation, Formal analysis, Data curation, Conceptualization. **Dow-Mu Koh:** Writing – review & editing, Writing – original draft, Visualization, Software, Resources, Project administration, Methodology, Investigation, Formal analysis, Data curation, Conceptualization. **Fiona McDonald:** Writing – review & editing, Writing – original draft, Visualization, Supervision, Resources, Project administration, Methodology, Investigation, Funding acquisition, Formal analysis, Data curation, Conceptualization. **Corrine Faivre-Finn:** Writing – review & editing, Writing – original draft, Visualization, Supervision, Resources, Project administration, Methodology, Investigation, Funding acquisition, Formal analysis, Data curation, Conceptualization.

## Declaration of competing interest

The authors declare that they have no known competing financial interests or personal relationships that could have appeared to influence the work reported in this paper.
