# Supplementary material for: Magnetic resonance imaging thoracic organ-at-risk atlas for radiation oncology
Source: Phys Imaging Radiat Oncol. 2026 Mar 21;38:100952. doi: 10.1016/j.phro.2026.100952 (PMC13053797; doi:10.1016/j.phro.2026.100952)
Supplement: Supplementary Data 1 [file mmc1.docx]

**Supplementary Material**

| Sequence | Motion Management | TR/TE  (ms) | FOV  (mm^2^) | Acquired Voxel Size (mm^3^) | Bandwidth  (Hz/Px) | Acquisition  Time  (mm:ss) | Slices |
| --- | --- | --- | --- | --- | --- | --- | --- |
| T_1_w Radial GRE | Free breathing. Stack-of-stars radial acquisition | 3.18 / 1.57 | 320 x 320 | 1.25 x 1.25  x 3.5 | 630 | 6:04 | 96 |
| T_2_w TSE  (without fat saturation) | Free breathing.  Navigator triggered to exhale | 1800 / 80 | 320 x 320 | 1.25 x 1.25  x 3.5 | 260 | 6:14 – 13:04 | 96 |
| T_2_w Dixon TSE | Free breathing.  No motion management | 1800 / 91 | 384 x 384 | 1.0 x 1.0  x 3.0 | 725 | 6:09 | 54 |

**Supplementary Table S1:** Sequence acquisition details of MRI scans: T_1_w Radial GRE: volumetric T_1_-weighted radial stack-of-stars spoiled gradient echo with golden angle spacing; T_2_w TSE: T_2_-weighted turbo spin echo; T_2_w Dixon TSE: T_2_-weighted Dixon turbo-spin echo.

A
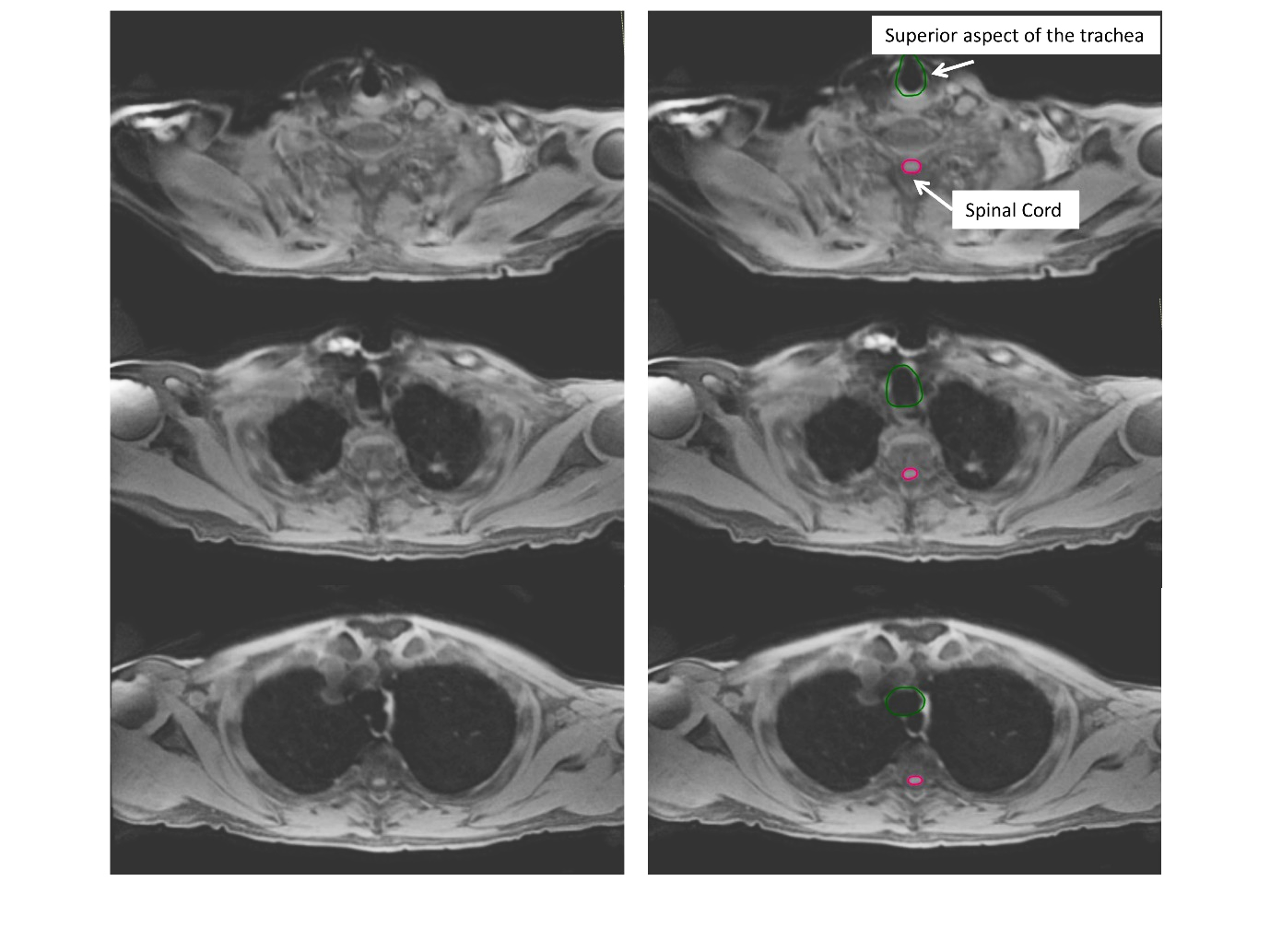


B
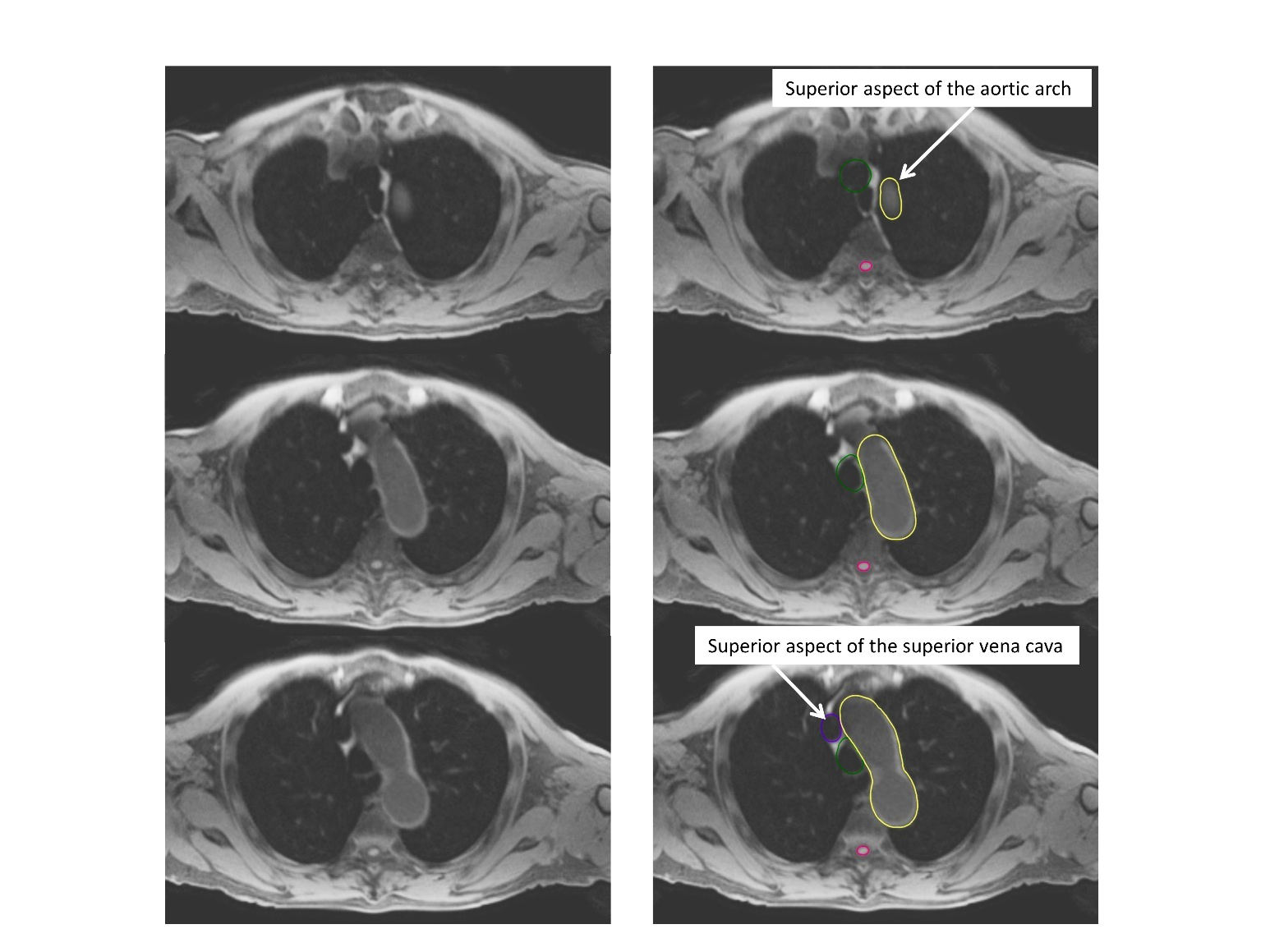


C
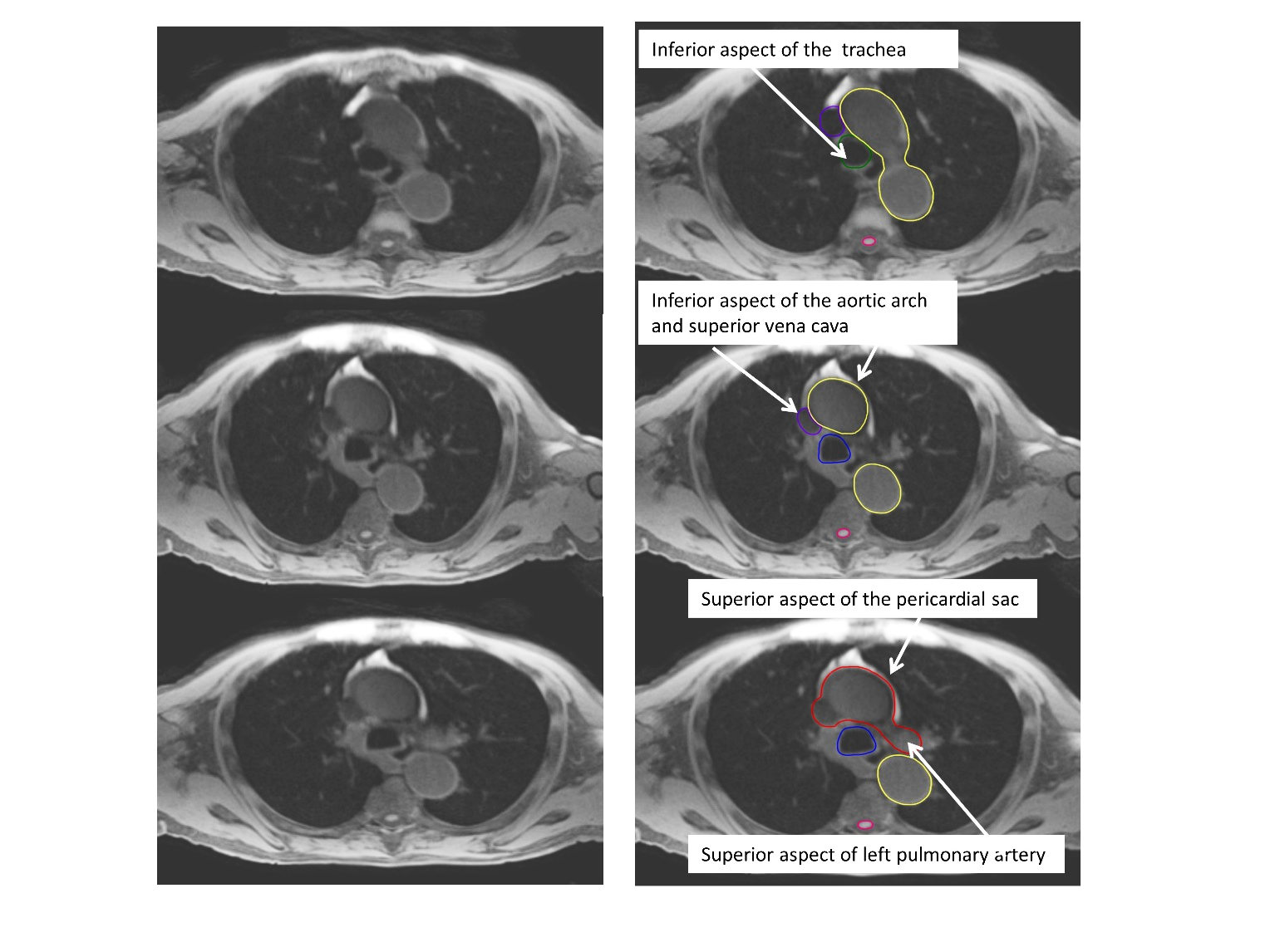


D
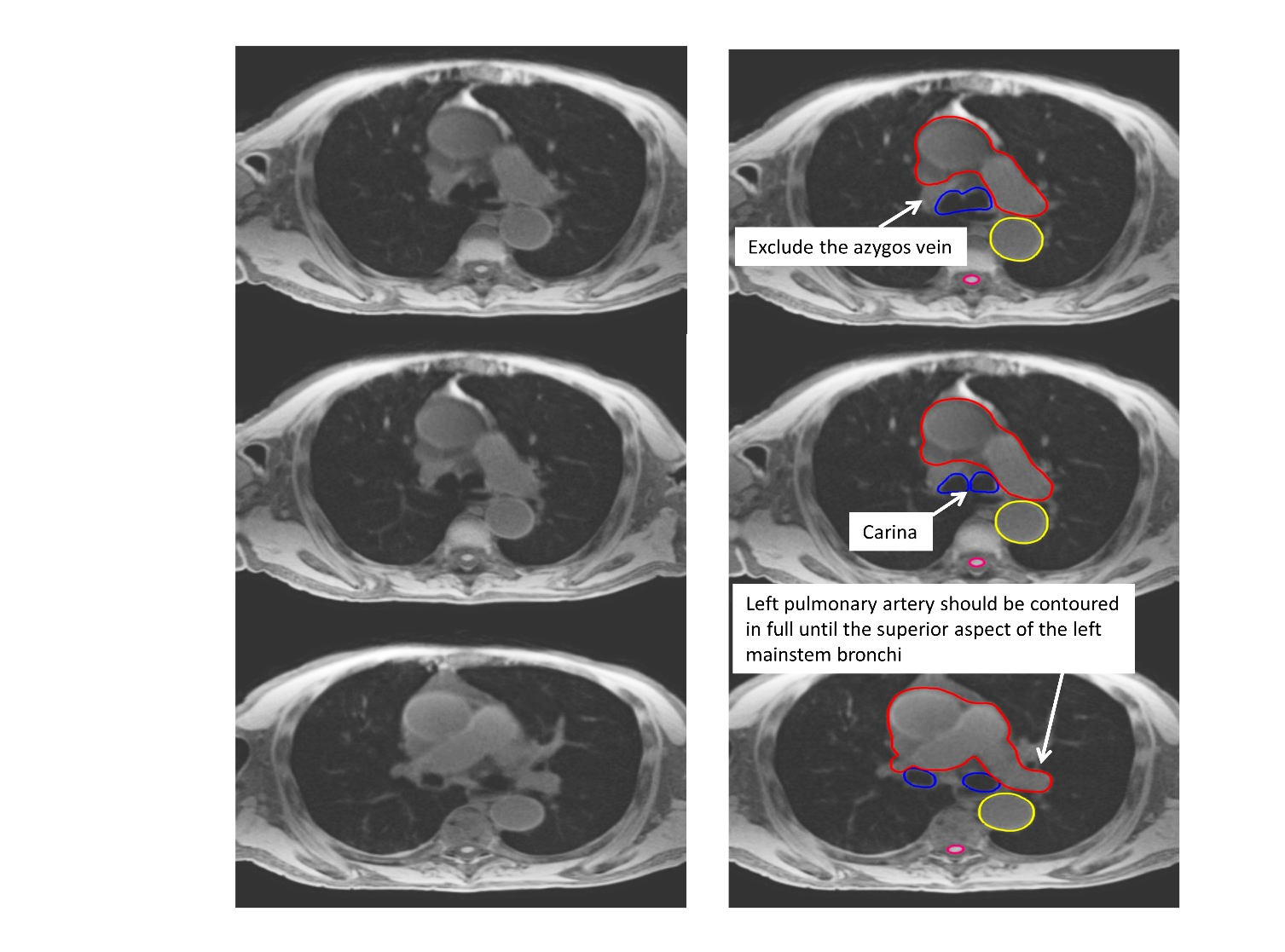


E
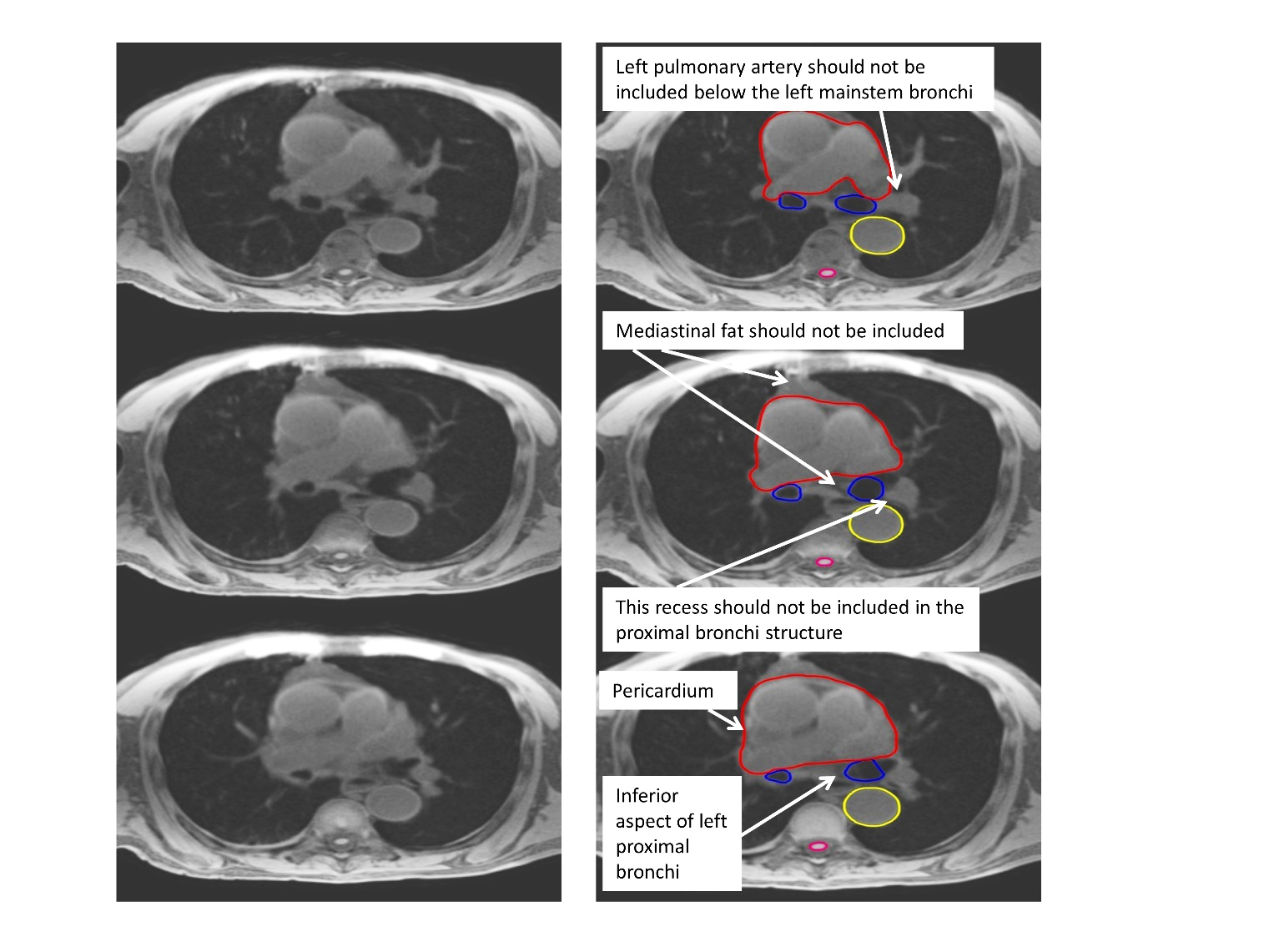


F
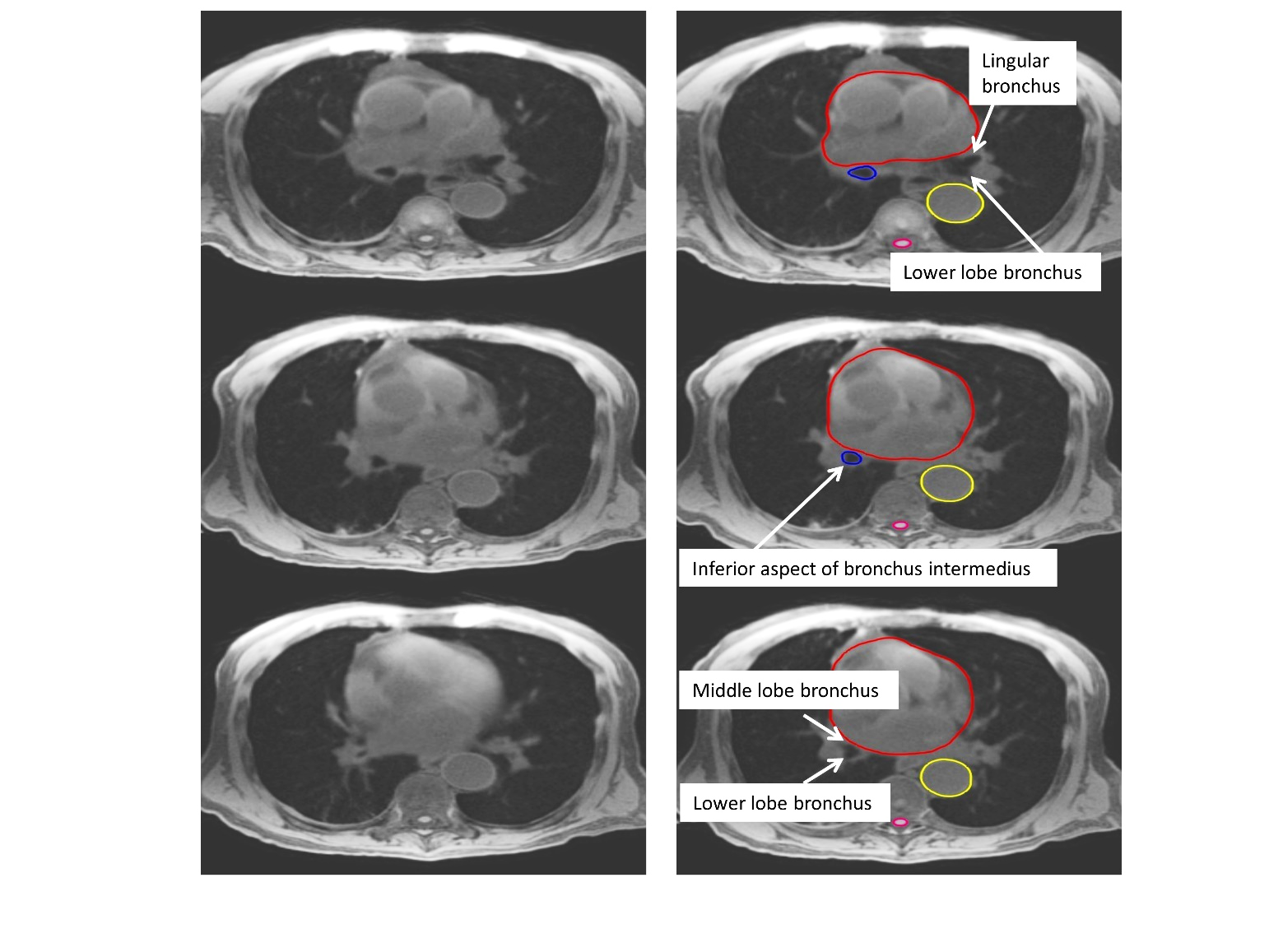


G
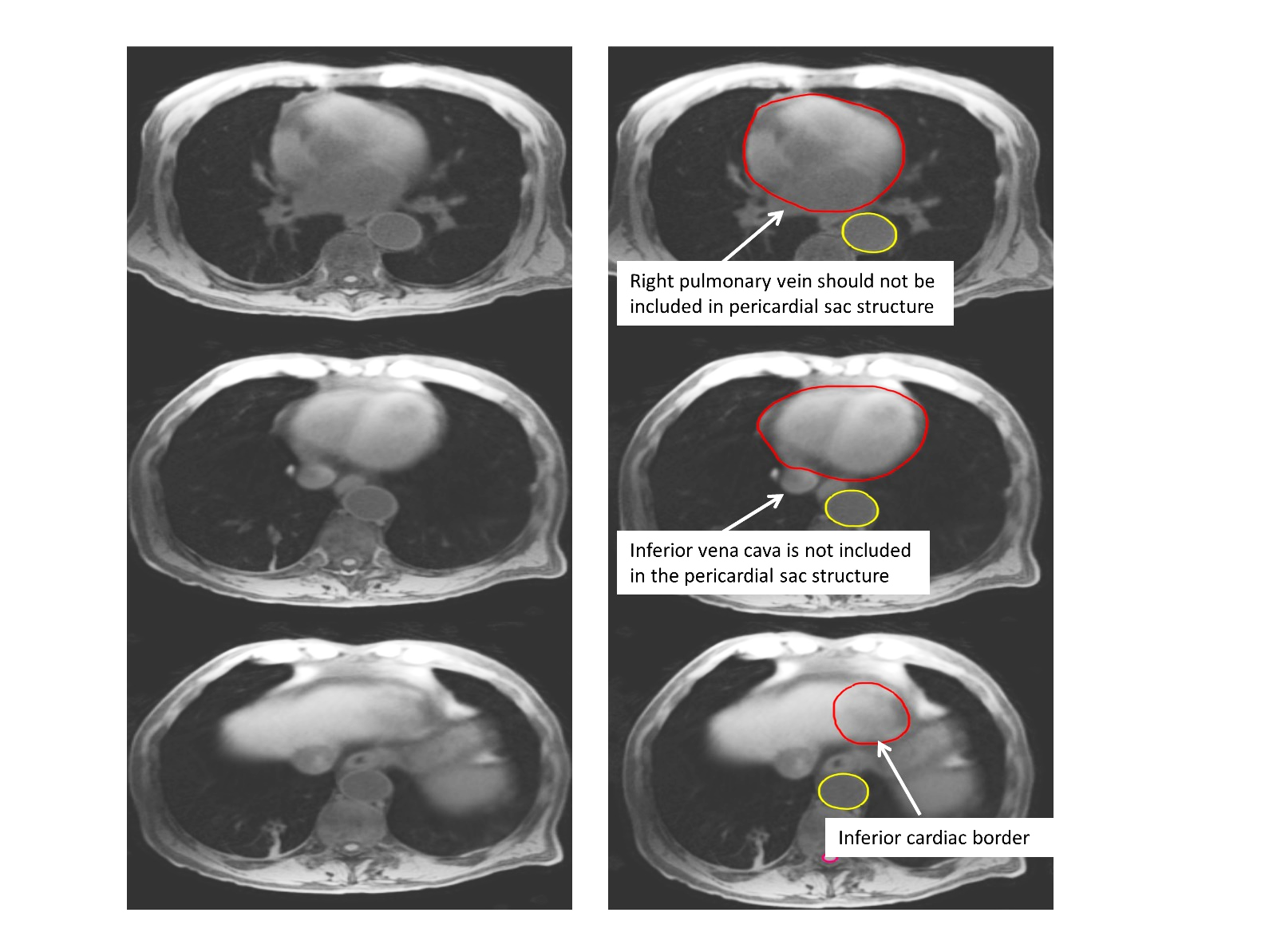


**Supplementary Figure S1**. Transverse T_1_w Radial GRE (volumetric stack-of-stars spoiled gradient echo with golden angle spacing) images; left: un-annotated, right: annotated and contoured; (A) from bottom of cricoid cartilage (B) from top of aortic arch (C) from aorto-pulmonary window (D) from carina (E) from left main bronchus (F) from left lower lobe bronchus (G) from pulmonary veins. Pink contour: Spinal cord; Blue contour: Proximal bronchi; Green contour: Trachea; Red contour: Pericardial sac; Yellow contour: Aorta; Purple contour: Superior vena cava.

A
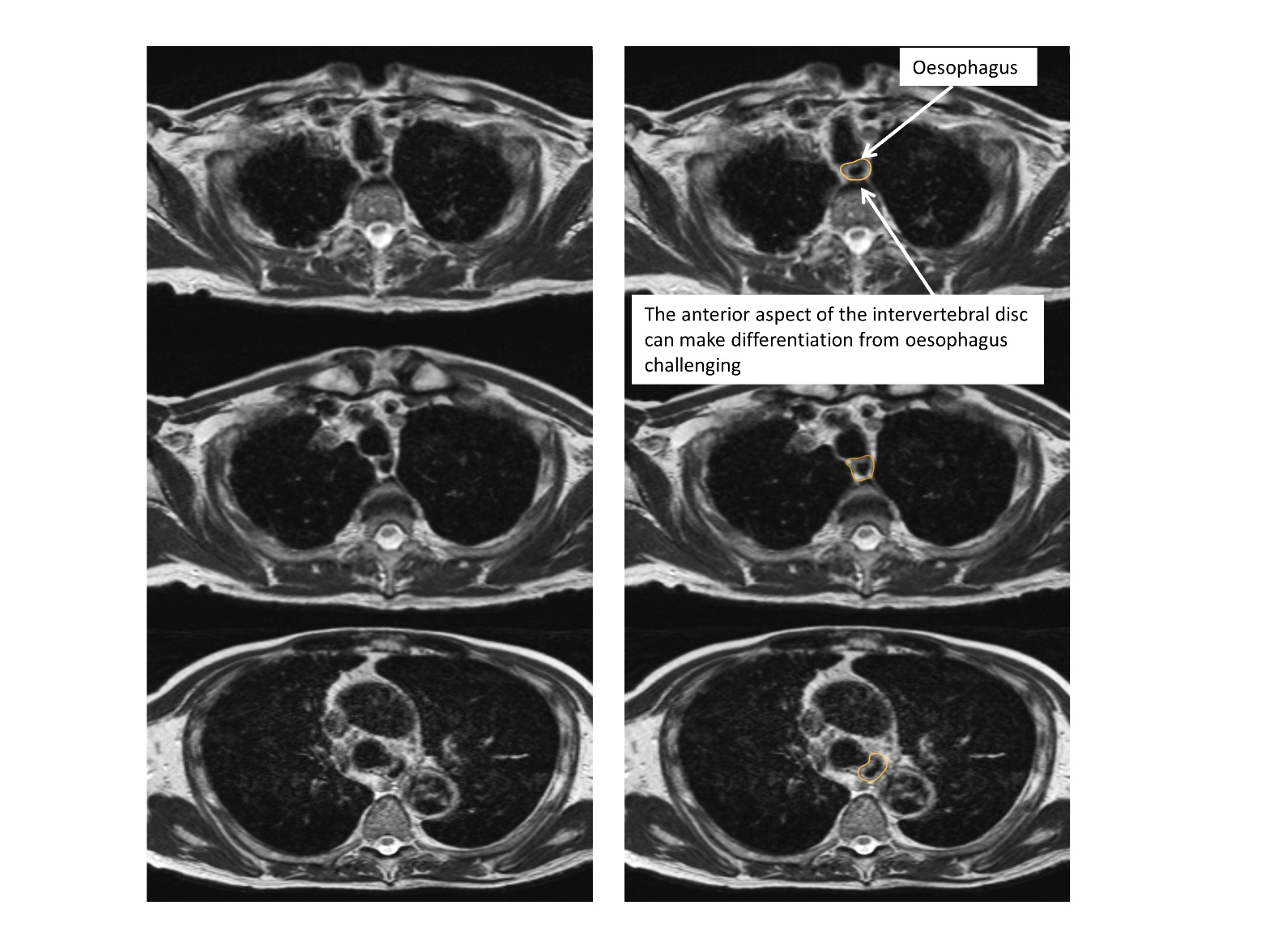


B
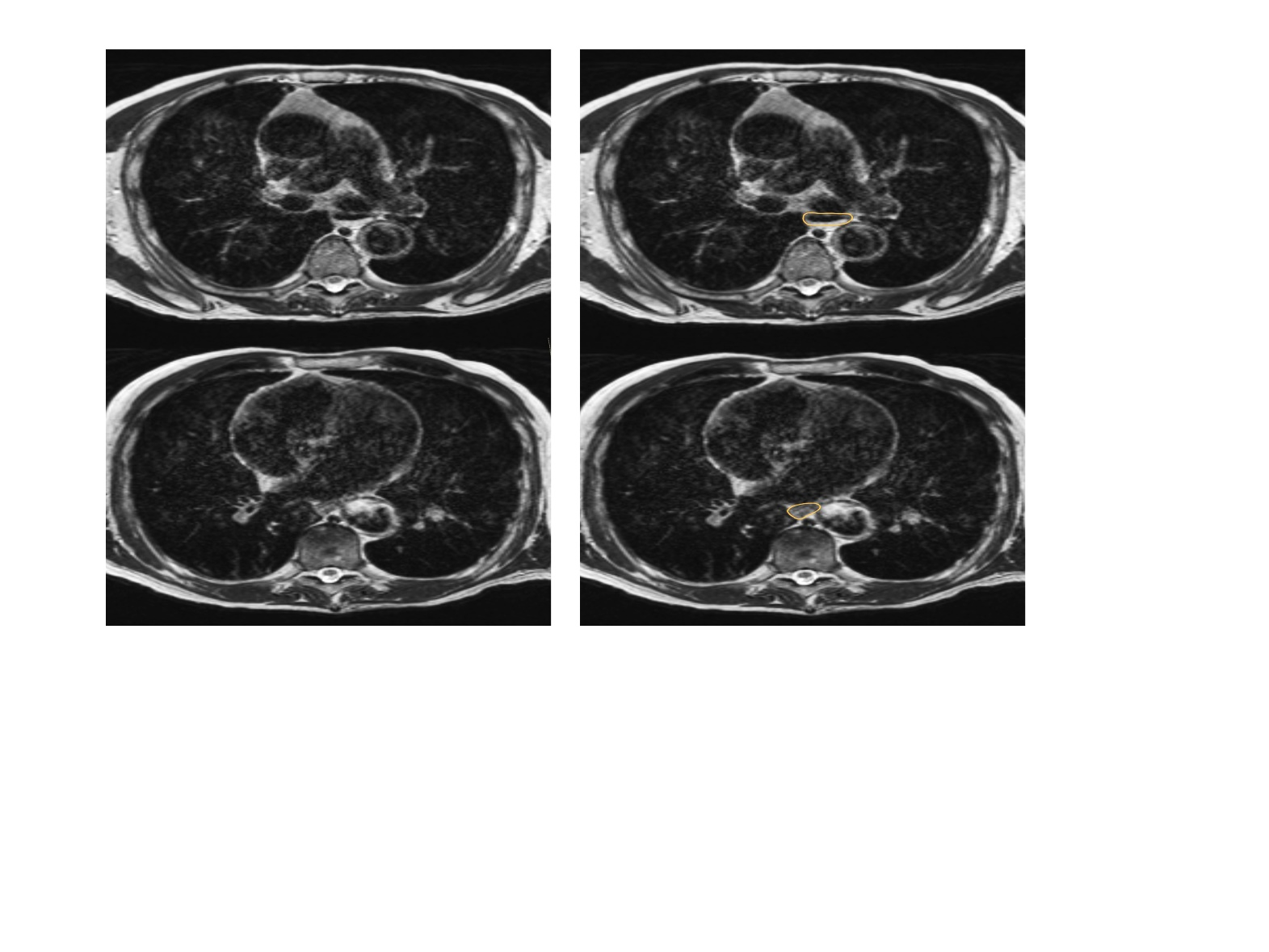


**Supplementary Figure S2.** Transverse T_2_w TSE (turbo spin echo) navigator triggered images of oesophagus (orange); left: un-annotated, right: annotated and contoured; (A) from clavicular heads, (B) from pulmonary artery bifurcation. Orange contour: Oesophagus.

**Supplementary Figure S3**. Illustration of brachial plexus anatomy indicating the position of the subclavian artery and vein relative to the brachial plexus, and the anatomical differentiation of the brachial plexus from roots to trunks, divisions, cords and terminal branches.

**T2w DIXON TSE ‘fat only’ T2w DIXON TSE ‘water only’**

A
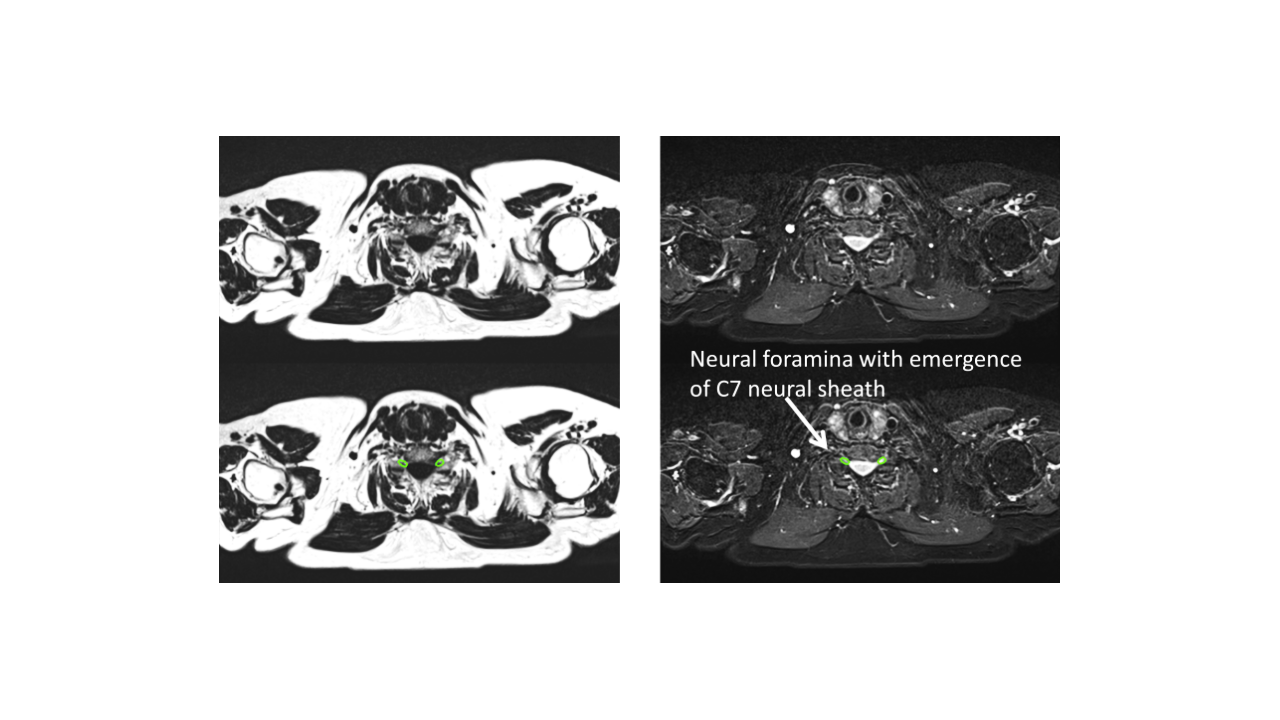


B
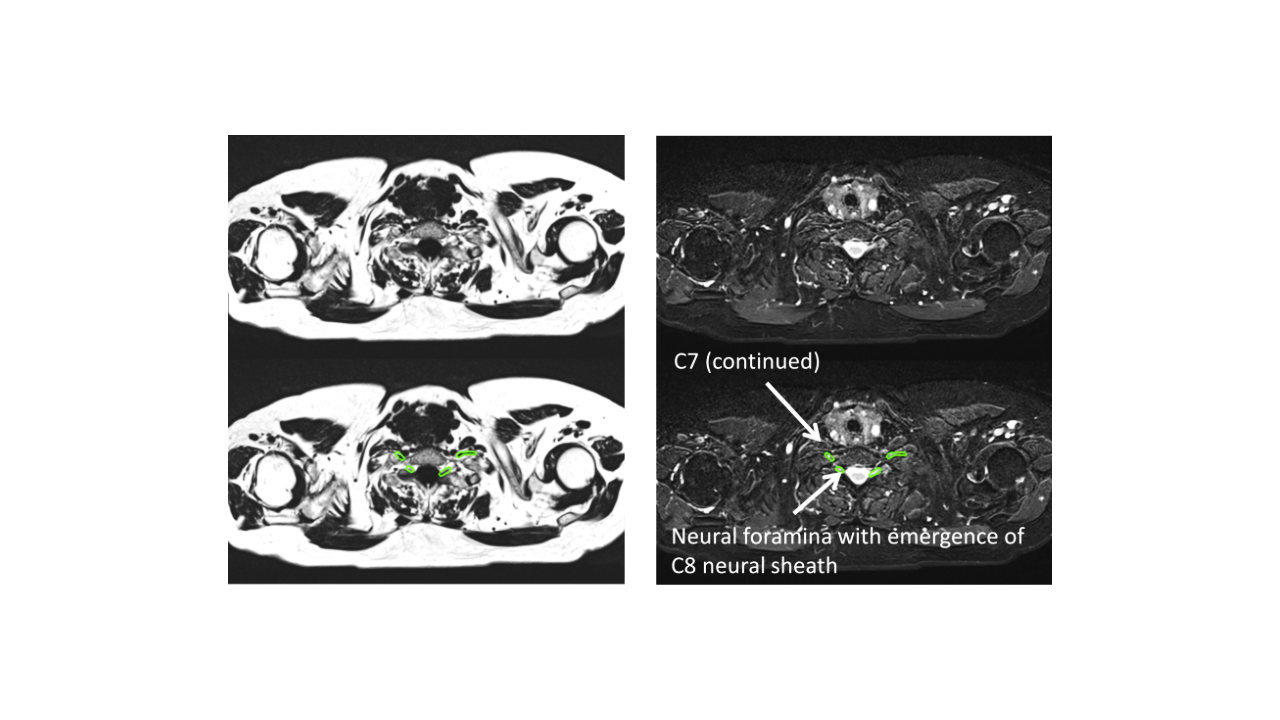


**T2w DIXON TSE ‘fat only’ T2w DIXON TSE ‘water only’**

C
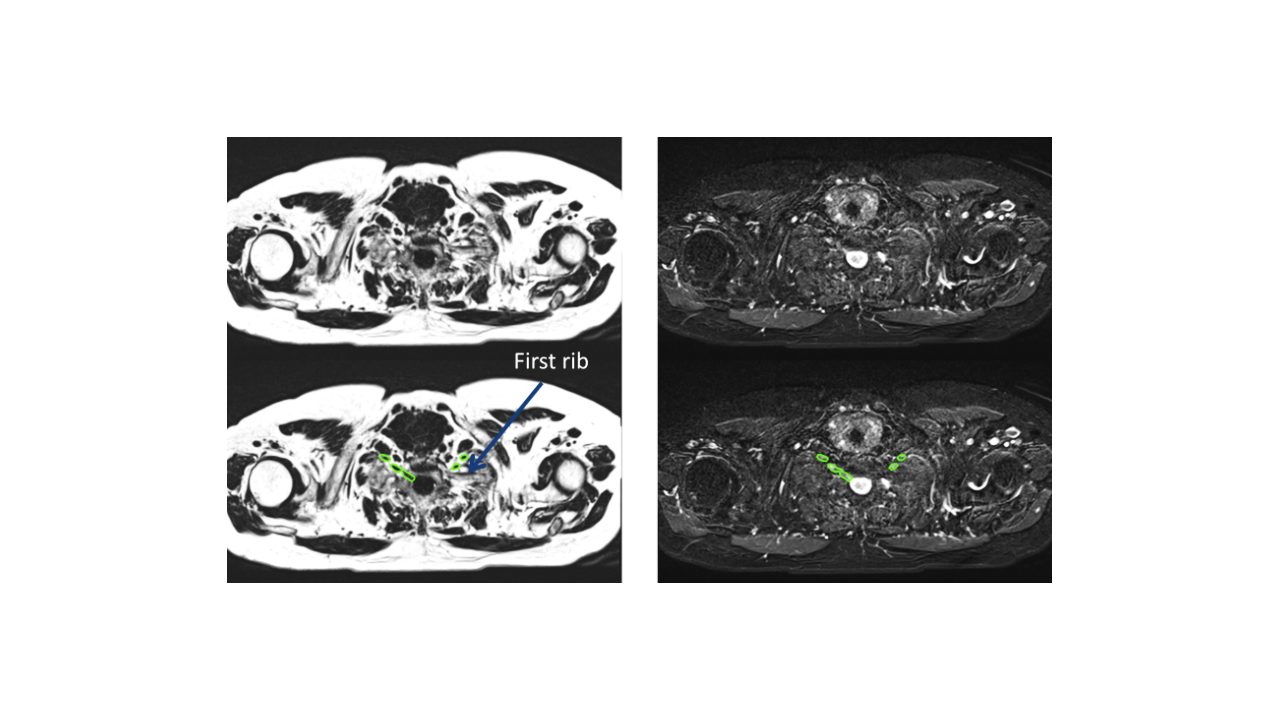


D
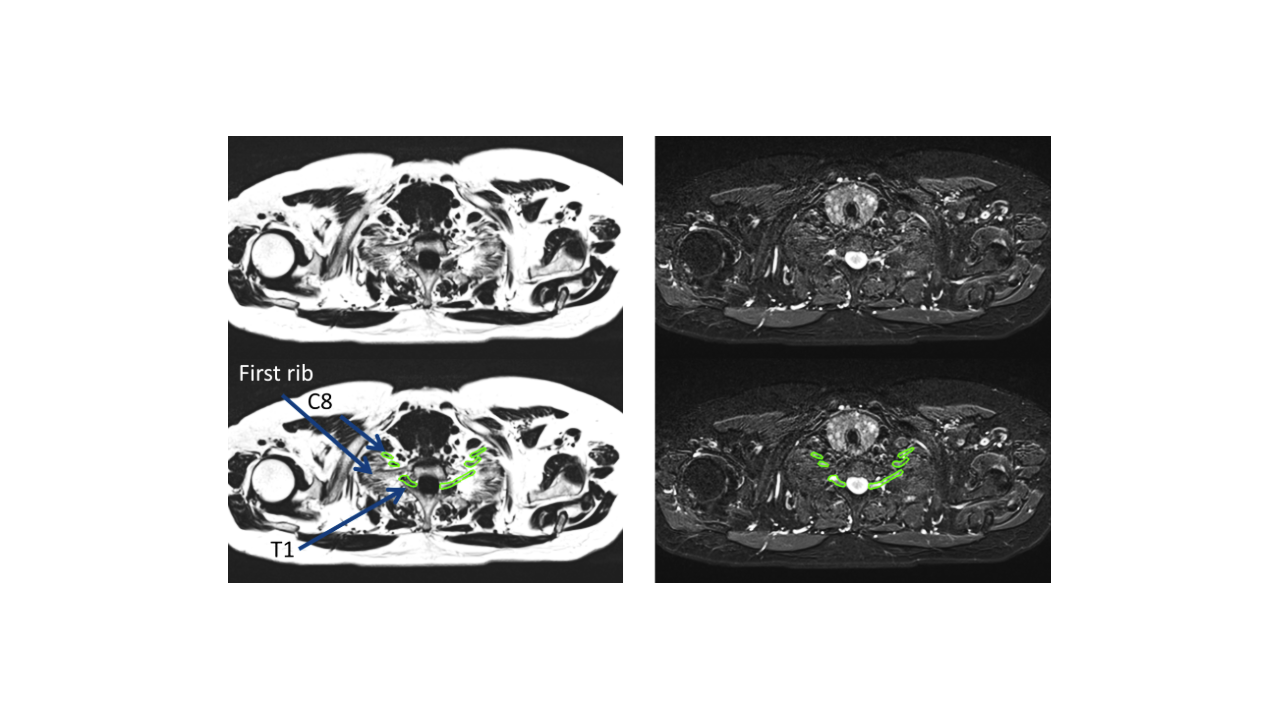


**Supplementary Figure S4**. Transverse T_2_w Dixon TSE (turbo-spin echo) images of brachial plexus; left: T_2_w Dixon TSE, fat only; right: T_2_w Dixon TSE, water only. The images are grouped in sets of four (A-D), top row un-annotated, bottom row annotated and contoured. Green contour: Brachial plexus envelope.
